# Impact of rapid response system in mortality and complications post-orthopedic surgery: a retrospective cohort study

**DOI:** 10.1186/s13741-024-00458-9

**Published:** 2024-10-04

**Authors:** Hey-ran Choi, In-Ae Song, Tak Kyu Oh

**Affiliations:** 1 Department of Anesthesiology and Pain Medicine, Inje University Sanggye Paik Hospital, Seoul, South Korea; 2 Department of Anesthesiology and Pain Medicine, Seoul National University Bundang Hospital, 173, Beon-gil, Bundang-gu, Seongnam, South Korea; 3 Department of Anesthesiology and Pain Medicine, College of Medicine, Seoul National University, Seoul, South Korea

**Keywords:** Postoperative complications, Critical care, Mortality, Orthopedic surgery, Rapid response system

## Abstract

**Background:**

Rapid response systems (RRSs) are used in hospitals to identify and treat deteriorating patients. However, RRS implementation and outcomes in orthopedic and surgical patients remain controversial. We aimed to investigate whether the RRS affects mortality and complications after orthopedic surgery.

**Methods:**

The National Health Insurance Service of South Korea provided the data for this population-based cohort study. Individuals who were admitted to the hospital that implemented RRS were categorized into the RRS group and those admitted to a hospital that did not implement the RRS were categorized into the non-RRS group. In-hospital mortality and postoperative complications were the endpoints.

**Results:**

A total of 931,774 adult patients were included. Among them, 93,293 patients underwent orthopedic surgery in a hospital that implemented RRS and were assigned to the RRS group, whereas 838,481 patients were assigned to the non-RRS group. In multivariable logistic regression analysis, the RRS group was not associated with in-hospital mortality after orthopedic surgery compared with the non-RRS group (odds ratio [OR] 0.93, 95% confidence interval [CI] 0.80, 1.08; *P* = 0.350). However, the RRS group was associated with a 14% lower postoperative complication rate after orthopedic surgery than the non-RRS group (OR 0.86, 95% CI 0.84, 0.86; *P* < 0.001).

**Conclusions:**

The RRS was not associated with in-hospital mortality following orthopedic surgery in South Korea. However, RRS deployment was related to a decreased risk of postoperative complications in patients undergoing orthopedic surgery.

**Supplementary Information:**

The online version contains supplementary material available at 10.1186/s13741-024-00458-9.

## Background

A rapid response system (RRS) is a specialized healthcare system employed by hospitals to promptly recognize and address the deteriorating condition of patients admitted to hospital wards (Lyons et al. 2018; Honarmand et al. 2024). To avoid cardiac arrest, trained intensivists can use the RRS to obtain alerts regarding abnormal vital signs ahead of time (Devita et al. 2006). Cardiac arrest and overall mortality in wards were significantly reduced after implementing the RRS (Maharaj et al. 2015; Jung et al. 2016).

The RRS was associated with reduced postoperative cardiac arrest in South Korea (Oh et al. 2018). However, the association between RRS implementation and outcomes among orthopedic and surgical patients remains controversial. Orthopedic surgery outcomes are important because the number of orthopedic procedures has increased owing to a rapidly aging society (Mastnak et al. 2022). However, only a few studies have focused on the impact of the RRS on outcomes in orthopedic patients (Ramos et al. 2020; Song et al. 2021; Zhang et al. 2021), but the effect of the RRS on mortality and complications after orthopedic surgery remains unelucidated. In addition, instead of comparing the effects of the RRS over the same period, previous studies utilized a before-and-after study design (Oh et al. 2018; Song et al. 2021).

Therefore, this study aimed to investigate whether the RRS affects mortality and complications after orthopedic surgery during the same period. We hypothesized that the RRS is associated with improved outcomes in orthopedic patients.

## Methods

### Study design, setting, ethical approval, and informed consent

This retrospective population-based cohort study was approved by the Institutional Review Board (IRB) (IRB approval number: X-2303–819-902). The sharing of data for this initiative was authorized by the Big Data Center of the National Health Insurance Service (NHIS) (NHIS-2023–1-526). Informed consent was not necessary for data analyses due to the retrospective nature of this study and the utilization of anonymized data.

### NHIS database

This research utilized information from the NHIS, South Korea’s sole public insurance system. All disease diagnoses and prescription information for any medication, procedure, or both must be entered into the NHIS database by law. Registration enables individuals to qualify for government-sponsored health insurance programs. Classifications from the 10th Revision of the International Classification of Diseases (ICD-10) were used to extract all diagnoses. The NHIS, a healthcare system operated by the South Korean government, mandates the registration of foreign residents who have been in the country for more than 6 months. Moreover, comprehensive data regarding the death dates and socioeconomic status of each individual can be found in the NHIS database (Lee et al. 2017).

### Study population

We included adult patients who were admitted to the hospital and underwent orthopedic surgery between January 1, 2019, and December 31, 2021, in South Korea. The orthopedic procedures are detailed in Table S1. Orthopedic procedures were classified into four groups: total knee arthroplasty (TKA), total hip arthroplasty (THA), fracture surgery, and other arthroplasties. Only initial orthopedic surgery was included in the study if it was performed more than twice during the study period. By applying these inclusion criteria, we aimed to ensure that the patients included in our study had similar characteristics, thereby promoting homogeneity. Among the included patients, those who were admitted to the hospital that used the RRS were assigned to the RRS group, whereas those who were admitted to the hospital that did not operate the RRS were assigned to the non-RRS group.

### RRS in South Korea

South Korea’s Ministry of Health and Welfare has been paying insurance payments to hospitals that use the RRS since 2019 (Lee and Hong 2019). When a hospital establishes a separate rapid response team and offers monitoring or an RRS to patients in general wards, the “RRS operating charge” is calculated once each day of hospitalization. The RRS must be supported by experts in internal medicine, neurology, surgery, neurosurgery, thoracic surgery, anesthesiology, pain medicine, and emergency medicine. RRS nurses must have at least 3 years of clinical experience in a regular hospital emergency department or intensive care unit. A video laryngoscope, portable mechanical ventilator, portable ultrasonography device, and point-of-care testing device are required for the RRS operation. Type 1 RRS must be operational 24 h a day, 365 days a year; Type 2 must be operational for at least 5 days per week, 16 h per day; and Type 3 for at least 5 days per week, 8 h per day.

### Study endpoints

This study had two endpoints: in-hospital mortality and postoperative complications. In-hospital mortality was defined as death after orthopedic surgery during hospitalization. Postoperative complications were defined as the occurrence of the following diseases during hospitalization after orthopedic surgeries: cerebral infarction or hemorrhage (ICD-10 codes I60 to I64), acute coronary events (I21, I22, and I252), heart failure (I50), pulmonary embolism (I26), acute and subacute hepatic failure (K720), acute renal failure (N17), sepsis (A40 and A41), wound infection (T793 and T814), pneumonia (J12 to J18 and J69), and urinary tract infection (N390, T835, and N30). Categorization criteria for postoperative complications were based on previous research (Makito et al. 2020).

### Collected covariates

Demographic data, such as age and sex, were obtained. Employment status, household income level, and residence were collected as covariates to indicate the patients' socioeconomic status. Five categories of household income levels were developed, including the four quartile ratio groups and the medical aid program group. Individuals who were poor and unable to pay insurance premiums were classified as participants in the government medical aid programs. The capital and other important communities were classified as urban areas, while the remaining regions were classified as rural areas.

The Charlson Comorbidity Index and underlying disability were used to reflect the comorbidity status of the patients. Charlson Comorbidity Index scores at hospital admission were calculated using the ICD-10 codes (Table S2) registered in the NHIS database.

Furthermore, it is mandatory to register all disabilities in the NHIS database to determine eligibility for a diverse range of benefits provided by social welfare programs in South Korea. Every disability must be formally diagnosed by a medical professional, based on the challenges encountered during the execution of routine activities. In Table S3, the classification of disabilities is detailed. The severity of the condition determined which patient was assigned to one of six severity classifications (first: most severe; sixth: least severe). Grades one through three were deemed “severe,” whereas grades four through six were deemed “mild to moderate.”

To reflect hospital capacity, hospital level (A, B, C, and D), postoperative intensive care unit (ICU), duration of stay in the ward, type of anesthesia (general or regional), and year of surgery were collected as covariates. Duration of stay in the ward (days) was collected because the RRS targeted hospitalized patients in the ward (not the ICU).

### Methodology for statistical analysis

Clinicopathological characteristics, represented as categorical variables, are expressed as means and standard deviations, and categorical variables are expressed as numbers and percentages. To compare the clinicopathological characteristics of the RRS group with those of the non-RRS group, the *t*-test was used for continuous variables and the chi-square test was used for categorical variables.

A hierarchical approach was used to determine the hospital levels, which were included as covariates. Hierarchical cluster analysis was performed using hospital-related variables, such as hospital type (tertiary general, general, and other types of hospital), total number of general and specialist doctors, nurses, pharmacists, hospital beds, operating room, and adult ICU beds using agglomerative clustering. Four hospital levels were determined based on the hierarchical clustering analysis results. Table S4 provides information about the characteristics of the hospitals.

After adjusting for covariates, we used multivariable logistic regression to determine whether the RRS group had an increased risk of in-hospital mortality or postoperative complications compared with the non-RRS group. The adjustment model incorporated all covariates, and the outcomes were displayed as odds ratios (ORs) with 95% confidence intervals (CIs). In addition, we performed multivariable logistic regression analyses to examine whether the RRS group had postoperative complications. Sensitivity and subgroup analyses were conducted based on the type of RRS and surgery to determine whether these factors influenced the results. All statistical analyses were conducted using R software (version 4.0.3, R Utilities). The threshold for significance was set at *P* < 0.05.

## Results

### Study population

**Fig. 1:**
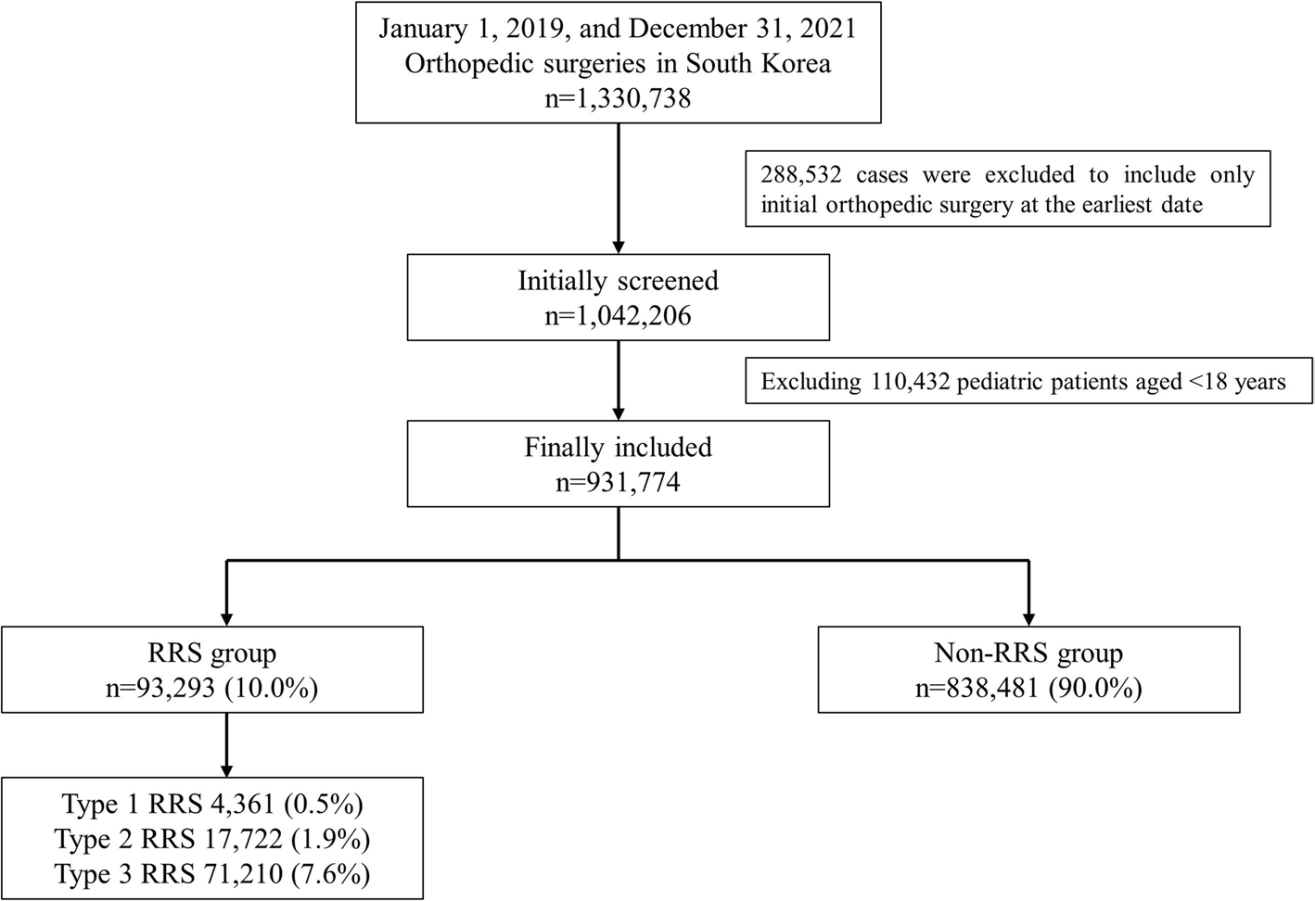
Flow chart depicting patient selection process. RRS, rapid response system

A total of 1,330,738 patients were hospitalized and underwent orthopedic surgeries, and 288,532 were excluded to include only those who underwent initial orthopedic surgery at the earliest date. Next, 1,042,206 patients were screened. After excluding 110,432 pediatric patients aged < 18 years, 931,774 adult patients were included in the study. Among them, 93,293 (10.0%) patients underwent orthopedic surgery in the RRS group, whereas 838,481 (90.0%) were assigned to the non-RRS group, as shown in Fig. 1. Table 1 shows the comparison of clinicopathological characteristics between the RRS and non-RRS groups.

**Table 1:** Comparison of clinicopathological characteristics between the RRS and non-RRS groups

Variable	RRS group *n* = 93,293	Non-RRS group *n* = 838,481	*P*-value
Age, year	65.6 (17.5)	59.4 (18.5)	< 0.001
Male sex	36,566 (39.2)	364,259 (43.4)	< 0.001
Having a job	46,835 (50.2)	458,700 (54.7)	< 0.001
Household income level			< 0.001
Q1 (lowest)	11,785 (12.6)	135,303 (16.1)	
Q2	11,193 (12.0)	135,998 (16.2)	
Q3	15,398 (16.5)	166,604 (19.9)	
Q4 (highest)	29,996 (32.2)	230,726 (27.5)	
Medical aid program	4434 (4.8)	42,002 (5.0)	
Unknown	20,487 (22.0)	127,848 (15.2)	
Residence			< 0.001
Urban area	37,425 (40.1)	299,712 (35.7)	
Rural area	55,868 (59.9)	538,769 (64.3)	
Underlying disability			< 0.001
Mild to moderate	10,143 (10.9)	62,919 (7.5)	
Severe	4391 (4.7)	24,075 (2.9)	
CCI, point	0.9 (1.5)	0.6 (1.1)	< 0.001
Postoperative ICU admission	3298 (3.5)	4347 (0.5)	< 0.001
Stay in ward, day	11.8 (8.4)	12.6 (11.6)	< 0.001
LOS, day	11.9 (8.6)	12.7 (11.6)	< 0.001
Total cost for hospitalization, USD	6821.2 (5,524.8)	3906.7 (4,014.1)	< 0.001
Regional anesthesia	27,807 (29.8)	339,050 (40.4)	< 0.001
Hospital level			< 0.001
Level A	18,444 (19.8)	0 (0.0)	
Level B	0 (0.0)	466,065 (55.6)	
Level C	61,272 (65.7)	59,312 (7.1)	
Level D	13,577 (14.6)	313,104 (37.3)	
Type of arthroplasty			< 0.001
TKA	27,218 (29.2)	199,542 (23.8)	
THA	20,971 (22.5)	37,395 (4.5)	
Fracture	43,916 (47.1)	599,430 (71.5)	
Other arthroplasty	1188 (1.3)	2114 (0.3)	
Year of surgery			0.256
2019	30,244 (32.4)	274,059 (32.7)	
2020	30,316 (32.5)	271,509 (32.4)	
2021	32,733 (35.1)	292,913 (34.9)	

RRS Rapid response system, *CCI* Charlson comorbidity index, *ICU* Intensive care unit, LOS Length of hospital stays, *USD* United States Dollars, *TKA* Total knee arthroplasty, *THA* Total hip arthroplasty

### In-hospital mortality and postoperative complications

Table 2 shows a comparison of the in-hospital mortality and postoperative complication rates between the RRS and non-RRS groups. The RRS group had a higher postoperative in-hospital mortality rate (0.3%; 315/93,293) than the non-RRS group (0.1%; 1252/838,481) (*P* < 0.001). The RRS group also showed a higher postoperative complication rate (14.3%; 13,341/93,293) than the non-RRS group (11.3%; 95,128/838,481) (*P* < 0.001).

**Table 2:** Comparison of the in-hospital mortality and postoperative complication rates between the RRS and non-RRS groups

Variable	RRS group *n* = 93,293	Non-RRS group *n* = 838,481	*P* value
In-hospital mortality	315 (0.3)	1252 (0.1)	< 0.001
90-day mortality	2093 (2.2)	6940 (0.8)	< 0.001
Postoperative complication	13,341 (14.3)	95,128 (11.3)	< 0.001
Cerebral infarction or hemorrhage	2790 (3.0)	9563 (1.1)	< 0.001
Acute coronary events	783 (0.8)	11,421 (1.4)	< 0.001
Heart failure	5666 (6.1)	31,807 (3.8)	< 0.001
Pulmonary embolism	1775 (1.9)	8401 (1.0)	< 0.001
Acute and subacute hepatic failure	22 (0.0)	353 (0.0)	0.007
Acute renal failure	1098 (1.2)	3476 (0.4)	< 0.001
Sepsis	884 (0.9)	5383 (0.6)	< 0.001
Wound infection	308 (0.3)	14,604 (1.7)	< 0.001
Pneumonia	2274 (2.4)	14,224 (1.7)	< 0.001
Urinary tract infection	1974 (2.1)	24,228 (2.9)	< 0.001

*RRS* Rapid response system

### Multivariable logistic regression modeling

Table 3 shows the results of the multivariable logistic regression models for in-hospital mortality and postoperative complications after orthopedic surgery. The RRS group was not associated with in-hospital mortality after orthopedic surgery, compared with the non-RRS group (OR 0.93, 95% CI 0.80, 1.08; *P* = 0.350; model 1). However, the RRS group was associated with a 14% lower postoperative complication rate after orthopedic surgery, compared with the non-RRS group (OR 0.86, 95% CI 0.84, 0.86; *P* < 0.001; model 2). The RRS group showed a lower association with heart failure (OR 0.90, 95% CI 0.86, 0.86; *P* < 0.001), pulmonary embolism (OR 0.93, 95% CI 0.87, 0.98; *P* = 0.038), pneumonia (OR 0.72, 95% CI 0.68, 0.76; *P* < 0.001), and urinary tract infections (OR 0.77, 95% CI 0.73, 0.81; *P* < 0.001). All ORs with 95% CIs in multivariable model 1 and model 2 are presented in Table S5 and Table S6, respectively.

**Table 3:** Multivariable logistic regression models for in-hospital mortality and postoperative complications after orthopedic surgery

Variable	OR (95% CI)	*P*-value
RRS group (vs non-RRS group)		
In-hospital mortality (model 1)	0.93 (0.80, 1.08)	0.350
Postoperative complication (model 2)	0.86 (0.84, 0.89)	< 0.001
Cerebral infarction or hemorrhage	1.05 (0.98, 1.11)	0.147
Acute coronary events	0.93 (0.84, 1.03)	0.173
Heart failure	0.90 (0.86, 0.94)	< 0.001
Pulmonary embolism	0.93 (0.87, 0.98)	0.038
Acute and subacute hepatic failure	0.87 (0.68, 1.12)	0.281
Acute renal failure	1.00 (0.94, 1.08)	0.913
Sepsis	1.10 (0.98, 1.20)	0.066
Pneumonia	0.72 (0.68, 0.76)	< 0.001
Urinary tract infection	0.77 (0.73, 0.81)	< 0.001

*OR* Odds ratio, *CI* Confidence interval, *RRS* Rapid response system

### Sensitivity and subgroup analyses

Table S7 shows the sensitivity analysis results according to the RRS type. Types 1 (OR 0.87, 95% CI 0.76, 0.98; *P* = 0.042), 2 (OR 0.57, 95% CI 0.53, 0.61; *P* < 0.001), and 3 (OR 0.91, 95% CI 0.88, 0.93; *P* < 0.001) RRSs were associated with lower postoperative complication rate, compared with the non-RRS group. Table 4 shows the subgroup analysis results according to surgery type. The RRS group showed a lower association of postoperative complication than the non-RRS group in the TKA (OR 0.67, 95% CI 0.63, 0.71; *P* < 0.001), THA (OR 0.94, 95% CI 0.89, 0.99; *P* = 0.038), and fracture surgery groups (OR 0.89, 95% CI 0.86, 0.93; *P* < 0.001).

**Table 4:** Subgroup analysis results according to surgery type

Variable	OR (95% CI)	*P*-value
RRS (vs non-RRS)		
TKA group		
In-hospital mortality	0.93 (0.47, 1.85)	0.834
Postoperative complication	0.67 (0.63, 0.71)	< 0.001
THA group		
In-hospital mortality	0.95 (0.73, 1.22)	0.667
Postoperative complication	0.94 (0.89, 0.99)	0.038
Fracture group		
In-hospital mortality	0.87 (0.71, 1.07)	0.190
Postoperative complication	0.89 (0.86, 0.93)	< 0.001
Other arthroplasty		
In-hospital mortality	0.25 (0.02, 3.94)	0.322
Postoperative complication	1.10 (0.75, 1.64)	0.622

*OR* Odds ratio, *CI* Confidence interval, *RRS* Rapid response system, *TKA* Total knee arthroplasty, *THA* Total hip arthroplasty

## Discussion

In this population-based cohort study conducted in South Korea, RRS implementation was not associated with in-hospital mortality in patients who underwent orthopedic surgery. However, postoperative complications after orthopedic surgery significantly decreased with the RRS implementation. This association is more evident in patients with heart failure, pulmonary embolism, pneumonia, and urinary tract infections.

There are several possible reasons for the no association between the RRS implementation status and in-hospital mortality compared to postoperative complications. As shown in Table 1, patients admitted to hospitals that implemented RRS were sicker than those who were admitted to non-RRS hospitals. Before adjusting the covariates, the RRS group showed a higher mortality rate than the non-RRS group. Although we adjusted several factors in the multivariable regression models for in-hospital mortality, there may be unmeasured and possible confounders that might have influenced the study results; the results might have been different if these unmeasured covariates were adjusted. Furthermore, many patients in the non-RRS group underwent treatment from “hospital level B” with limited medical facilities and support (Table S4). Given that more severely ill patients were admitted to hospitals that implemented the RRS and there was no significant difference in postoperative mortality between the RRS and non-RRS groups, more severely ill patients should be selected and transferred to better-equipped facilities for orthopedic surgeries.

Several studies have reported the effects of RRS in surgical patients (Oh et al. 2018; Sento et al. 2021; Song et al. 2020). However, previous studies have focused on overall postoperative patients (Oh et al. 2018; Sento et al. 2021; Song et al. 2020), and information regarding the specific effects of RRS on postoperative outcomes among orthopedic patients is lacking. A previous study in a single institution reported that the RRS implementation enabled early notice and rapid intervention in deteriorating patients following hip fracture surgery, resulting in shorter hospital stays (Song et al. 2021). Another study reported that RRS could be beneficial for improving postoperative outcomes in patients who underwent THA (Kaplan et al. 2020). In addition to these previous literature (Song et al. 2021; Kaplan et al. 2020), our study offers the advantage of analyzing a large nationwide cohort population simultaneously rather than using a before-and-after study design.

In the present study, RRS implementation was associated with a lower risk of heart failure after orthopedic surgery. A previous prospective cohort study conducted in the United Kingdom reported a 5% risk of inpatient heart failure as a postoperative complication (Roche et al. 2005). Prevention of heart failure is critical because it is a major risk factor for increased mortality following noncardiac surgeries (Lerman et al. 2019). Moreover, acute myocardial infarction and associated myocardial dysfunction are the most common causes of heart failure (Cahill and Kharbanda 2017), and the RRS may have recognized early electrocardiogram changes in patients hospitalized in the ward after orthopedic surgery, allowing for prompt coronary intervention.

This study found that the RRS was associated with postoperative pulmonary embolism in orthopedic patients. Pulmonary embolisms are a critical problem in orthopedic patients. The incidence of fatal pulmonary embolism varies from 0.19% to 3.4% after total hip or knee replacement surgery (Westrich et al. 2000; Lawton et al. 2003), and the incidence of asymptomatic pulmonary embolism was reported to be 12% (Freedman et al. 2000). Mortality associated with pulmonary embolism after major surgery has been reported as 16.9–31% (Temgoua et al. 2017). Postoperative pulmonary embolism can be prevented by mechanical and pharmacological interventions (Nisio et al. 2016). The RRS usually detects clinical deterioration, such as hypoxemia, rapid respiratory rate, and low oxygen saturation, which reflect signs of pulmonary embolism. The RRS substantially contributes to averting the progression of patients with suspected pulmonary embolism by promptly diagnosing and initiating timely treatment.

Furthermore, the OR for postoperative complications after orthopedic surgery in the RRS group was the lowest for postoperative pneumonia. The overall incidence of postoperative pneumonia after hip fracture surgery is reported to be 5% in the older population (Lee and Kim 2022). Moreover, the incidence of postoperative pneumonia after TKA or THA is reported to be 0.3% (Ally et al. 2021). Postoperative pneumonia is a known risk factor for increased postoperative mortality in orthopedic patients (Jamali et al. 2018). Many clinical signs, such as fever, rapid respiratory rate, and low oxygen saturation can be detected early by the control tower of the RRS (Song et al. 2020). Thus, the RRS may have prevented the progression of pneumonia by prescribing antibiotics, suctioning sputum, and encouraging breathing during the early stages of pneumonia.

In addition, postoperative urinary tract infection is another major issue because it can significantly increase the risk of wound and deep periprosthetic joint infections after TKA or THA (Schmitt et al. 2020). High-grade fever related to urinary tract infection can be detected early by the control tower of the RRS. The RRS could intervene in patients by prescribing proper antibiotics and removing the urinary catheter to prevent the progression of urinary tract infection after orthopedic surgery.

This study had some limitations. First, although the RRS monitors all patients admitted to the ward, it remains unclear how many patients were treated with the RRS in this study. Second, some unmeasured and potential confounders may have influenced the study outcomes. Third, important parameters, such as body mass index, smoking history, and operative time were not adjusted for in this study because the data were not available in the NHIS database. Finally, while we controlled for hospital levels (four groups), we did not assess medical equipment, doctor-to-patient ratios, nurse-to-patient ratios, or the quality of medical staff in the hospital.

## Conclusions

In conclusion, the RRS was not associated with in-hospital mortality after orthopedic surgery. However, RRS implementation was associated with a lower risk of postoperative complications, such as heart failure, pulmonary embolism, pneumonia, and urinary tract infection, in patients who underwent orthopedic surgery. Our results suggest that the RRS may be beneficial for the prevention of postoperative complications in orthopedic patients.

## Supplementary Information

Supplementary Material 1: Table S1. Code of orthopedic surgery in this study

Supplementary Material 2: Table S2. The ICD-10 codes used by comorbidity to compute the Charlson comorbidity index

Supplementary Material 3: Table S3. Classification of disabilities in South Korea

Supplementary Material 4: Table S4. Hospital characteristics

Supplementary Material 5: Table S5. All ORs with 95% CIs in multivariable model 1

Supplementary Material 6: Table S6. All ORs with 95% CIs in multivariable model 2

Supplementary Material 7: Table S7. Sensitivity analysis results according to RRS type

## Data Availability

The datasets used and analyzed during the current study are available from the corresponding author (TKO) upon reasonable request.

## Abbreviations

CI: Confidence interval
ICD-10: 10Th Revision of the International Classification of Diseases
NHIS: National Health Insurance Service
ICU: Intensive care unit
IRB: Institutional Review Board
OR: Odds ratio
RRS: Rapid Response Systems
TKA: Total knee arthroplasty
THA: Total hip arthroplasty
