# Supplementary material for: Impact of rapid response system in mortality and complications post-orthopedic surgery: a retrospective cohort study
Source: Perioper Med (Lond). 2024 Oct 4;13:98. doi: 10.1186/s13741-024-00458-9 (PMC11452942; doi:10.1186/s13741-024-00458-9)
Supplement: Supplementary file 1 — Supplementary Material 1: Table S1. Code of orthopedic surgery in this study [file 13741_2024_458_MOESM1_ESM.docx]

Table S1. Code of orthopedic surgery in this study

| **Op code** | **Op name** |
| --- | --- |
| N0711 | Total Arthroplasty [Hip] |
| N1711 | Revision of Total Arthroplasty [Hip] |
| N1714 | Revision of Total Arthroplasty [Finger,Toe] |
| N1715 | Revision of Hemiarthroplasty [Hip] |
| N1717 | Revision of Hemiarthroplasty [Finger,Toe] |
| N1721 | Revision of Total Arthroplasty [Hip] |
| N1724 | Revision of Total Arthroplasty [Finger,Toe] |
| N1725 | Revision of Hemiarthroplasty [Hip] |
| N2070 | Total Arthroplasty [Hip] |
| N2072 | Replacement Arthroplasty-Total [Knee] |
| N2075 | Replacement Arthroplasty-Total [Ankle] |
| N2077 | Replacement Arthroplasty-Total [Knee] |
| N2079 | Replacement Arthroplasty-Total [Ankle] |
| N2710 | Hemiarthroplasty [Hip] |
| N2711 | Replacement Arthroplasty-Hemiarhtroplasty [Shoulder] |
| N2712 | Replacement Arthroplasty-Hemiarhtroplasty [Knee] |
| N2715 | Replacement Arthroplasty-Hemiarhtroplasty [Ankle] |
| N2717 | Replacement Arthroplasty-Hemiarhtroplasty [Knee] |
| N2719 | Replacement Arthroplasty-Hemiarhtroplasty [Ankle] |
| N3710 | Revision of Total Arthroplasty [Hip] |
| N3712 | Revision of Total Arthroplasty [Knee] |
| N3715 | Revision of Total Arthroplasty [Ankle] |
| N3717 | Revision of Total Arthroplasty [Knee] |
| N3719 | Revision of Total Arthroplasty [Ankle] |
| N3720 | Revision of Total Arthroplasty [Hip] |
| N3722 | Revision of Total Arthroplasty [Knee] |
| N3725 | Revision of Total Arthroplasty [Ankle] |
| N3727 | Revision of Total Arthroplasty [Knee] |
| N3729 | Revision of Total Arthroplasty [Ankle] |
| N4710 | Revision of Hemiarthroplasty [Hip] |
| N4712 | Revision of hemiarthroplasty [Knee] |
| N4715 | Revision of hemiarthroplasty[Ankle] |
| N4717 | Revision of hemiarthroplasty [Knee] |
| N4719 | Revision of hemiarthroplasty [Ankle] |
| N4720 | Revision of Hemiarthroplasty [Hip] |
| N4722 | Revision of hemiarthroplasty [Knee] |
| N4725 | Revision of hemiarthroplasty [Ankle] |
| N4727 | Revision of hemiarthroplasty [Knee] |
| N4729 | Revision of hemiarthroplasty [Ankle] |
| N0592 | Open Reduction of Fracture and Dislocation of Spine or Pelvis-Acetabulum |
| N0593 | Open Reduction of Fracture and Dislocation of Spine or Pelvis-Pelvis |
| N0601 | Open Reduction of Fractured Extremity [Femur] |
| N0604 | Open Reduction of Fractured Extremity [Clavicle,Patella,Carpal Bone,Tarsal Bone] |
| N0605 | Open Reduction of Fractured Extremity [Metacarpal,Metatarsal,Finger,Toe] |
| N0606 | Closed Pinning [Metacarpal,Metatarsal,Finger,Toe] |
| N0611 | Open Reduction of Fractured Extremity [Femur] |
| N0614 | Open Reduction of Fractured Extremity [Clavicle,Patella,Carpal Bone,Tarsal Bone] |
| N0615 | Open Reduction of Fractured Extremity [Metacarpal,Metatarsal,Finger,Toe] |
| N0641 | Closed Reduction of Fractured Extremity [Pelvis,Femur] |
| N0645 | Closed Reduction of Fractured Extremity [Metacarpal,Metatarsal,Finger,Toe] |
| N0991 | Closed Pinning [Femur] |
| N0999 | Open Reduction of Fractured Extremity (Closed Pinning)-Crus(Tibia) |
| N1000 | Open Reduction of Fractured Extremity (Closed Pinning)-Crus(Fibula) |
| N1001 | Open Reduction of Fractured Extremity (Closed Pinning)-Crus(Tibia and Fibula) |
| N1604 | Open Reduction of Fractured Extremity-Crus (Tibia) |
| N1605 | Open Reduction of Fractured Extremity-Crus (Fibula) |
| N1606 | Open Reduction of Fractured Extremity-Crus (Tibia and Fibula) |
| N1614 | Open Reduction of Fractured Extremity-Crus (Tibia) |
| N1615 | Open Reduction of Fractured Extremity-Crus (Fibula) |
| N1616 | Open Reduction of Fractured Extremity-Crus (Tibia and Fibula) |
