# Supplementary material for: Impact of rapid response system in mortality and complications post-orthopedic surgery: a retrospective cohort study
Source: Perioper Med (Lond). 2024 Oct 4;13:98. doi: 10.1186/s13741-024-00458-9 (PMC11452942; doi:10.1186/s13741-024-00458-9)
Supplement: Supplementary file 3 — Supplementary Material 3: Table S3. Classification of disabilities in South Korea [file 13741_2024_458_MOESM3_ESM.docx]

Table S3. Classification of disabilities in South Korea

| Type of disability | | Subcategory |
| --- | --- | --- |
|  | Physical disability | Amputation disorder, joint disorder, physical dysfunction, and deformity |
|  | Brain lesion disability | Complex disorders due to brain damage |
|  | Visual disturbance | Blindness, visual impairment |
|  | Hearing disability | Hearing impairment, equilibrium dysfunction |
|  | Speech disability | Language disorder, voice disorder, speech disorder |
|  | Intellectual disorder | IQ is below 70 |
|  | Autism | Autistic disorders such as childhood autism |
|  | Mental disorder | Schizophrenia, schizoaffective disorder, bipolar affective disorder, recurrent depressive disorder |
|  | Renal disorder | Treated by dialysis or have had a kidney transplant |
|  | Heart disorder | Cardiac dysfunction that significantly restricts daily life |
|  | Respiratory disability | Chronic, severe respiratory dysfunction that significantly restricts daily life |
|  | Hepatopathy | Chronic, severe liver function abnormalities that significantly restrict daily life |
|  | Facial disfigurement | Disorders caused by deformities such as abstraction, depression, and thickening of the facial area |
|  | Intestinal and urinary fistulae | Stoma and urostomy that significantly restrict daily life |
|  | Epilepsy | Chronic, severe epilepsy that significantly restricts daily life |
