# Supplementary material for: Impact of rapid response system in mortality and complications post-orthopedic surgery: a retrospective cohort study
Source: Perioper Med (Lond). 2024 Oct 4;13:98. doi: 10.1186/s13741-024-00458-9 (PMC11452942; doi:10.1186/s13741-024-00458-9)
Supplement: Supplementary file 4 — Supplementary Material 4: Table S4. Hospital characteristics [file 13741_2024_458_MOESM4_ESM.docx]

Table S4. Hospital characteristics

| Variable | | Hospital level A  n=5 | Hospital level B  n=96,196 | Hospital level C  n=63 | Hospital level D  n=1,438 |
| --- | --- | --- | --- | --- | --- |
| Type of hospital | |  |  |  |  |
|  | Tertiary general hospital | 5 (100.0) | 0 (0.0) | 38 (60.3) | 43 (0.0) |
|  | General hospital | 0 | 24 (0.0) | 25 (39.7) | 368 (0.4) |
|  | Other type of hospital | 0 | 96,172 (100.0) | 0 (0.0) | 97,291 (99.6) |
| Total number of doctor | | 1,307.4 (298.9) | 0.7 (1.4) | 332.6 (111.2) | 14.4 (26.1) |
| Total number of specialist doctor | | 716.4 (150.6) | 0.6 (1.3) | 182.7 (57.0) | 12.2 (17.9) |
| Total number of nurse | | 2,085.4 (294.1) | 2.2 (5.3) | 715.9 (233.1) | 67.9 (71.6) |
| Total number of pharmacist | | 117.2 (22.6) | 0.2 (0.6) | 23.8 (11.5) | 1.3 (2.0) |
| Total number of hospital beds | | 1,940.0 (515.4) | 4.1 (17.6) | 817.8 (177.0) | 244.8 (88.8) |
| Total number of OR beds | | 52.0 (15.8) | 0.1 (0.5) | 15.9 (5.1) | 1.6 (2.5) |
| Total number of adult ICU beds | | 125.6 (27.5) | 0.0 (0.2) | 50.7 (18.1) | 3.2 (7.7) |
| Total number of ER beds | | 57.2 (24.7) | 0.0 (0.5) | 34.4 (10.0) | 4.2 (7.3) |

OR, operating room; ICU, intensive care unit; ER, emergency room
