# Supplementary material for: Impact of rapid response system in mortality and complications post-orthopedic surgery: a retrospective cohort study
Source: Perioper Med (Lond). 2024 Oct 4;13:98. doi: 10.1186/s13741-024-00458-9 (PMC11452942; doi:10.1186/s13741-024-00458-9)
Supplement: Supplementary file 5 — Supplementary Material 5: Table S5. All ORs with 95% CIs in multivariable model 1 [file 13741_2024_458_MOESM5_ESM.docx]

Table S5. All ORs with 95% CIs in multivariable model 1

| Variable | | OR (95% CI) | *P*-value |
| --- | --- | --- | --- |
| Age, year | | 1.10 (1.09, 1.10) | <0.001 |
| Male sex | | 1.66 (1.49, 1.86) | <0.001 |
| Having a job | | 0.81 (0.72, 0.92) | 0.001 |
| Household income level | |  |  |
|  | Q1 (lowest) | 1 |  |
|  | Q2 | 0.91 (0.74, 1.11) | 0.336 |
|  | Q3 | 0.96 (0.80, 1.15) | 0.659 |
|  | Q4 (highest) | 0.82 (0.69, 0.96) | 0.016 |
|  | Medical aid program | 1.27 (1.04, 1.55) | 0.019 |
|  | Unknown | 1.11 (0.91, 1.36) | 0.293 |
| Residence | |  |  |
|  | Urban area | 1 |  |
|  | Rural area | 1.33 (1.18, 1.50) | <0.001 |
| Underlying disability | |  |  |
|  | Mild to moderate | 1.13 (0.97, 1.30) | 0.109 |
|  | Severe | 2.09 (1.79, 2.46) | <0.001 |
| CCI, point | | 1.36 (1.33, 1.39) | <0.001 |
| Regional anesthesia (vs GA) | | 1.25 (1.11, 1.40) | <0.001 |
| Postoperative ICU admission | | 6.66 (5.82, 7.63) | <0.001 |
| Stay in ward, day | | 0.99 (0.99, 0.99) | 0.023 |
| Hospital level | |  |  |
|  | Level A | 1 |  |
|  | Level B | 0.80 (0.51, 1.25) | 0.334 |
|  | Level C | 1.83 (1.22, 2.75) | 0.003 |
|  | Level D | 2.12 (1.39, 3.24) | <0.001 |
| Type of arthroplasty | |  |  |
|  | TKA | 1 |  |
|  | THA | 5.34 (4.22, 6.75) | <0.001 |
|  | Fracture | 4.93 (3.97, 6.12) | <0.001 |
|  | Other arthroplasty | 3.05 (1.12, 8.34) | 0.029 |
| Year of surgery | |  |  |
|  | 2019 | 1 |  |
|  | 2020 | 0.98 (0.87, 1.12) | 0.791 |
|  | 2021 | 0.93 (0.82, 1.06) | 0.263 |

OR, odds ratio; CI, confidence interval; CCI, Charlson comorbidity index; GA, general anesthesia; ICU, Intensive care unit; TKA, total hip arthroplasty; THA, total hip arthroplasty
