# Supplementary material for: Impact of rapid response system in mortality and complications post-orthopedic surgery: a retrospective cohort study
Source: Perioper Med (Lond). 2024 Oct 4;13:98. doi: 10.1186/s13741-024-00458-9 (PMC11452942; doi:10.1186/s13741-024-00458-9)
Supplement: Supplementary file 6 — Supplementary Material 6: Table S6. All ORs with 95% CIs in multivariable model 2 [file 13741_2024_458_MOESM6_ESM.docx]

Table S6. All ORs with 95% CIs in multivariable model 2

| Variable | | OR (95% CI) | *P*-value |
| --- | --- | --- | --- |
| Age, year | | 1.04 (1.04, 1.04) | <0.001 |
| Male sex | | 1.03 (1.01, 1.05) | 0.001 |
| Having a job | | 0.99 (0.97, 1.01) | 0.198 |
| Household income level | |  |  |
|  | Q1 (lowest) | 1 |  |
|  | Q2 | 0.99 (0.97, 1.02) | 0.525 |
|  | Q3 | 0.97 (0.95, 1.00) | 0.035 |
|  | Q4 (highest) | 0.92 (0.90, 0.95) | <0.001 |
|  | Medical aid program | 1.04 (1.00, 1.07) | 0.051 |
|  | Unknown | 0.84 (0.81, 0.86) | <0.001 |
| Residence | |  |  |
|  | Urban area | 1 |  |
|  | Rural area | 1.21 (1.19, 1.23) | <0.001 |
| Underlying disability | |  |  |
|  | Mild to moderate | 1.08 (1.06, 1.11) | <0.001 |
|  | Severe | 1.21 (1.17, 1.26) | <0.001 |
| CCI, point | | 1.90 (1.89, 1.91) | <0.001 |
| Regional anesthesia (vs GA) | | 1.36 (1.34, 1.38) | <0.001 |
| Postoperative ICU admission | | 2.75 (2.60, 2.90) | <0.001 |
| Stay in ward, day | | 1.03 (1.03, 1.03) | <0.001 |
| Hospital level | |  |  |
|  | Level A | 1 |  |
|  | Level B | 1.42 (1.32, 1.53) | <0.001 |
|  | Level C | 2.47 (2.30, 2.65) | <0.001 |
|  | Level D | 2.22 (2.06, 2.39) | <0.001 |
| Type of arthroplasty | |  |  |
|  | TKA | 1 |  |
|  | THA | 1.76 (1.72, 1.81) | <0.001 |
|  | Fracture | 1.46 (1.44, 1.49) | <0.001 |
|  | Other arthroplasty | 1.29 (1.16, 1.44) | <0.001 |
| Year of surgery | |  |  |
|  | 2019 | 1 |  |
|  | 2020 | 1.01 (0.99, 1.03) | 0.230 |
|  | 2021 | 1.07 (1.05, 1.09) | <0.001 |

OR, odds ratio; CI, confidence interval; CCI, Charlson comorbidity index; GA, general anesthesia; ICU, Intensive care unit; TKA, total hip arthroplasty; THA, total hip arthroplasty
