# Supplementary material for: Impact of rapid response system in mortality and complications post-orthopedic surgery: a retrospective cohort study
Source: Perioper Med (Lond). 2024 Oct 4;13:98. doi: 10.1186/s13741-024-00458-9 (PMC11452942; doi:10.1186/s13741-024-00458-9)
Supplement: Supplementary file 7 — Supplementary Material 7: Table S7. Sensitivity analysis results according to RRS type [file 13741_2024_458_MOESM7_ESM.docx]

Table S7. Sensitivity analysis results according to RRS type

| Variable | | OR (95% CI) | *P*-value |
| --- | --- | --- | --- |
|  |  | RRS group (vs non-RRS group) |  |
| In-hospital mortality | |  |  |
|  | Type 1 RRS | 0.99 (0.49, 2.00) | 0.973 |
|  | Type 2 RRS | 0.69 (0.46, 1.05) | 0.085 |
|  | Type 3 RRS | 0.95 (0.81, 1.12) | 0.545 |
| Postoperative complication | |  |  |
|  | Type 1 RRS | 0.87 (0.76, 0.98) | 0.042 |
|  | Type 2 RRS | 0.57 (0.53, 0.61) | <0.001 |
|  | Type 3 RRS | 0.91 (0.88, 0.93) | <0.001 |

OR, odds ratio; CI, confidence interval; RRS, rapid response system
